# Bis{2-amino-2-oxo-*N*-[(1*E*)-1-(pyridin-2-yl-κ*N*)ethyl­idene]acetohydrazidato-κ^2^ *N*′,*O* ^1^}nickel(II)

**DOI:** 10.1107/S1600536812014109

**Published:** 2012-04-06

**Authors:** Cheikh Hamidou Kane, Ibrahima Elhadj Thiam, Farba Bouyagui Tamboura, Mohamed Gaye, Pascal Retailleau

**Affiliations:** a Département de Chimie, Faculté des Sciences et Techniques, Université Cheikh Anta Diop, Dakar, Senegal; b Centre de Recherche de Gif sur Yvette, Institut de Chimie des Substances Naturelles, UPR2301-CNRS, 1 Avenue la Terrasse, 91198 Gif sur Yvette cédex, France

## Abstract

In the title compound, [Ni(C_9_H_9_N_4_O_2_)_2_], the Ni^II^ ion is situated on a twofold rotation axis and is coordinated by two O and four N atoms from two tridentate {2-amino-2-oxo-*N*-[(1*E*)-1-(pyridin-2-yl-κ*N*)ethyl­idene]acetohydrazidate ligands in a distorted octa­hedral geometry. In the crystal, N—H⋯O and N—H⋯N hydrogen bonds link the mol­ecules into columns in [001]. The porous crystal packing is further stabilized *via* π–π inter­actions between the pyridine rings of neighbouring mol­ecules [centroid–centroid distance = 3.746 (3) Å] with voids of 270 Å^3^.

## Related literature

For the structures of related nickel complexes, see: Dieng *et al.* (2004 ▶); Tamboura *et al.* (2009 ▶); Mikuriya *et al.* (1996 ▶).
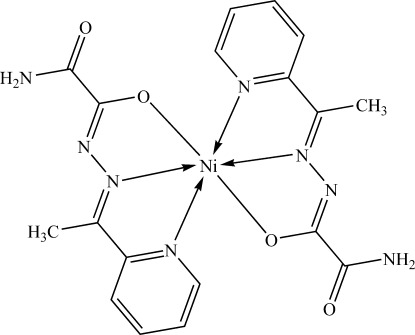


## Experimental

### 

#### Crystal data

[Ni(C_9_H_9_N_4_O_2_)_2_]
*M* *_r_* = 469.11
Monoclinic, 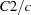

*a* = 16.703 (3) Å
*b* = 17.878 (4) Å
*c* = 8.929 (2) Å
β = 114.915 (5)°
*V* = 2418.2 (9) Å^3^
*Z* = 4
Mo *K*α radiation
μ = 0.84 mm^−1^
*T* = 293 K
0.21 × 0.14 × 0.13 mm

#### Data collection

Nonius KappaCCD diffractometer
Absorption correction: multi-scan (*SCALEPACK*; Otwinowski & Minor, 1997 ▶) *T*_min_ = 0.778, *T*_max_ = 0.897
6288 measured reflections
1781 independent reflections
1285 reflections with *I* > 2σ(*I*)
*R*_int_ = 0.046
θ_max_ = 23.5°

#### Refinement

*R*[*F* ^2^ > 2σ(*F* ^2^)] = 0.051
*wR*(*F* ^2^) = 0.137
*S* = 1.04
1777 reflections
142 parameters
H-atom parameters constrained
Δρ_max_ = 0.33 e Å^−3^
Δρ_min_ = −0.30 e Å^−3^

### 

Data collection: *DENZO* (Otwinowski & Minor, 1997 ▶) and *COLLECT* (Nonius, 1999 ▶); cell refinement: *DENZO* and *COLLECT*; data reduction: *SCALEPACK* (Otwinowski & Minor, 1997 ▶); program(s) used to solve structure: *SHELXS97* (Sheldrick, 2008 ▶); program(s) used to refine structure: *SHELXL97* (Sheldrick, 2008 ▶) and *CRYSTALBUILDER* (Welter, 2006 ▶); molecular graphics: *PLATON* (Spek, 2009 ▶); software used to prepare material for publication: *publCIF* (Westrip, 2010 ▶).

## Supplementary Material

Crystal structure: contains datablock(s) I, global. DOI: 10.1107/S1600536812014109/cv5274sup1.cif

Structure factors: contains datablock(s) I. DOI: 10.1107/S1600536812014109/cv5274Isup2.hkl

Additional supplementary materials:  crystallographic information; 3D view; checkCIF report

## Figures and Tables

**Table 1:** Hydrogen-bond geometry (Å, °)

*D*—H⋯*A*	*D*—H	H⋯*A*	*D*⋯*A*	*D*—H⋯*A*
N4—H4*A*⋯O2^i^	0.86	2.22	2.976 (5)	147
N4—H4*B*⋯N3^ii^	0.86	2.25	3.074 (5)	160

Symmetry codes: (i) 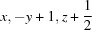; (ii) 
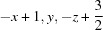
.

**Fig. 1.**
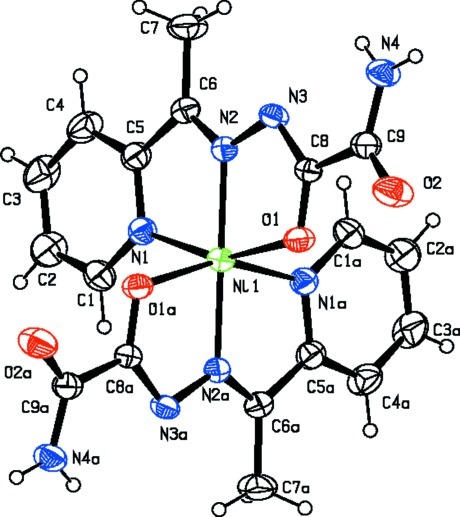
An ORTEP view of the title compound, showing the atom-numbering scheme [symmetry code: (a) -x + 1, y, -z + 1/2]. Displacement ellipsoids are plotted at the 30% probability level.

**Fig. 2.**
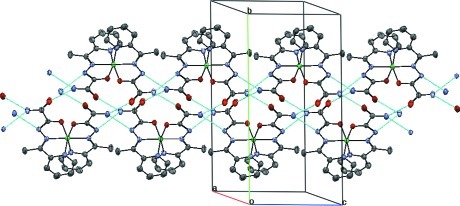
A portion of the crystal packing viewed down the a axis and showing hydrogen bonds as cyan lines.

## supplementary crystallographic information

### Comment

In the title compound (Fig. 1), the Ni^II^ ion is situated on a twofold rotational and adopts a distorted octahedral geometry. The coordination of the hydrazones to Ni center results in the formation of two five-membered chelating rings. In the two rings, the Ni–N_pyridyl_ bond lengths of 2.094 (3) Å are larger than those for Ni–N_iminol_ bonds [1.980 (3) Å]. The Ni–O1 involving the hydrazonic oxygen have the metal-ligand distance of 2.074 (3) Å. The Ni—N and Ni—O bond distances are similar to those observed in other mononuclear Ni^II^ complexes with similar tridentate ligands (Dieng *et al.*, 2004; Tamboura *et al.*, 2009). The following bond C6–N2 is not altered in the complex and remains with double bond character. The bond C8–N3 which was simple in character becomes a double bond after deprotonation of the N–H function. The N_imino_–Ni–O1 and N_pyridyl_–Ni–N_imino_ angles are 78.25 (13)° and 92.04 (13)° respectively. The deviation from 90° of the bond angles involving the chelation observed is presumably due to the formation of five-membered ring (Mikuriya *et al.*, 1996).

In the crystal structure, the intermolecular hydrogen-bonding network involving the acetamide groups and also the N3 atom (Table 1), propagates parallel to the crystallographic *c* axis (Fig.2). This contributes to display a double inverted *X* molecular pattern in the *ab* plane stabilized by π – π stacking interactions between adjacent pyridine rings with the centroid-centroid distance of 3.746 (3) Å.

### Experimental

2-Amino-2-oxo-*N*^'^-(1-(pyridin-2-yl)ethylidene)acetohydrazide (0.206 g, 1 mmol) was dissolved in 10 ml of ethanol and the LiOH (0.042 g, 2 mmol) was added with thorough shaking. To the resulting solution, Ni(CH_3_COO)_2_.4H_2_O (0.2489 g, 1 mmol) was added. Immediate change of the colour was observed. The mixture was stirred at room temperature during 2 h. The solution was filtered off and concentrated to tenth. Crystals that separated from the brown solution were filtered off and recrystallized in ethanol. On standing for three weeks, suitable X-rays crystals were obtained. Yield: 73.5%. Anal. Calc. for [C_18_H_18_N_8_O_4_Ni] (%): C, 46.09; H, 3.87; N, 23.89. Found: C, 46.06; H, 3.85; N, 23.87. Selected IR data (cm^-1^, KBr pellet): 3214, 1728, 1645, 1585, 1459, 768.

### Refinement

All H atoms were located in difference maps. They were then treated as riding in geometrically idealized positions, with C—H = 0.93 (aryl), or 0.96 Å(CH_3_) and N—H = 0.86 Å, and with *U*_iso_(H)=k*U*_eq_(C,*N*), where k = 1.5 for the methyl groups, and 1.2 for all other H atoms. Four low-resolution reflections were omitted due to beamstop shading (*OMIT* instruction in *SHELX97*-*L*)). Infinite cylindrical channels of 8 Å diameters ran through the crystal packing along the crystallographic *c* axis at positions *x*=0, *y*=1/2, *z* and *x*=1/2, *y*=0, *z* accounting for voids of 270 Å^3^ per unit cell but no solvent contribution to the X-ray diffraction was found.

### Crystal data

**Table:**

[Ni(C_9_H_9_N_4_O_2_)_2_]	*F*(000) = 968
*M**_r_* = 469.11	*D*_x_ = 1.289 Mg m^−^^3^
Monoclinic, *C*2/*c*	Mo *K*α radiation, λ = 0.71070 Å
Hall symbol: -C 2yc	Cell parameters from 1780 reflections
*a* = 16.703 (3) Å	θ = 0.4–23.5°
*b* = 17.878 (4) Å	µ = 0.84 mm^−^^1^
*c* = 8.929 (2) Å	*T* = 293 K
β = 114.915 (5)°	Block, brown
*V* = 2418.2 (9) Å^3^	0.21 × 0.14 × 0.13 mm
*Z* = 4	

### Data collection

**Table:**

Nonius KappaCCD diffractometer	1781 independent reflections
Radiation source: fine-focus sealed tube, Nonius Kappa CCD	1285 reflections with *I* > 2σ(*I*)
Graphite monochromator	*R*_int_ = 0.046
Detector resolution: 9 pixels mm^-1^	θ_max_ = 23.5°, θ_min_ = 2.3°
phi and ω scans	*h* = −18→18
Absorption correction: multi-scan (*SCALEPACK*; Otwinowski & Minor, 1997)	*k* = −18→20
*T*_min_ = 0.778, *T*_max_ = 0.897	*l* = −9→9
6288 measured reflections	

### Refinement

**Table:**

Refinement on *F*^2^	Primary atom site location: structure-invariant direct methods
Least-squares matrix: full	Secondary atom site location: difference Fourier map
*R*[*F*^2^ > 2σ(*F*^2^)] = 0.051	Hydrogen site location: difference Fourier map
*wR*(*F*^2^) = 0.137	H-atom parameters constrained
*S* = 1.04	*w* = 1/[σ^2^(*F*_o_^2^) + (0.0757*P*)^2^] where *P* = (*F*_o_^2^ + 2*F*_c_^2^)/3
1777 reflections	(Δ/σ)_max_ < 0.001
142 parameters	Δρ_max_ = 0.33 e Å^−^^3^
0 restraints	Δρ_min_ = −0.30 e Å^−^^3^

### Special details

**Table:**

Geometry. All e.s.d.'s (except the e.s.d. in the dihedral angle between two l.s. planes) are estimated using the full covariance matrix. The cell e.s.d.'s are taken into account individually in the estimation of e.s.d.'s in distances, angles and torsion angles; correlations between e.s.d.'s in cell parameters are only used when they are defined by crystal symmetry. An approximate (isotropic) treatment of cell e.s.d.'s is used for estimating e.s.d.'s involving l.s. planes.
Refinement. Refinement of *F*^2^ against ALL reflections. The weighted *R*-factor *wR* and goodness of fit *S* are based on *F*^2^, conventional *R*-factors *R* are based on *F*, with *F* set to zero for negative *F*^2^. The threshold expression of *F*^2^ > σ(*F*^2^) is used only for calculating *R*-factors(gt) *etc*. and is not relevant to the choice of reflections for refinement. *R*-factors based on *F*^2^ are statistically about twice as large as those based on *F*, and *R*- factors based on ALL data will be even larger. Five low-resolution reflections were omitted due to beamstop shading (*OMIT* instruction in *SHELX97*-*L*).

### Fractional atomic coordinates and isotropic or equivalent isotropic displacement parameters (Å^2^)

**Table:**

	*x*	*y*	*z*	*U*_iso_*/*U*_eq_	
Ni1	0.5000	0.30602 (4)	0.2500	0.0392 (3)	
O1	0.41866 (19)	0.38561 (16)	0.2833 (3)	0.0451 (8)	
O2	0.3304 (2)	0.4879 (2)	0.3757 (4)	0.0722 (11)	
N1	0.5999 (2)	0.22513 (19)	0.3236 (4)	0.0438 (9)	
N2	0.5383 (2)	0.30400 (18)	0.4922 (4)	0.0368 (8)	
N3	0.4949 (2)	0.35081 (19)	0.5577 (4)	0.0406 (9)	
N4	0.3876 (3)	0.4413 (2)	0.6353 (4)	0.0572 (11)	
H4A	0.3576	0.4710	0.6676	0.069*	
H4B	0.4232	0.4094	0.7031	0.069*	
C1	0.6314 (3)	0.1867 (3)	0.2321 (6)	0.0567 (13)	
H1	0.6064	0.1948	0.1187	0.068*	
C2	0.6990 (4)	0.1356 (3)	0.2970 (7)	0.0708 (16)	
H2	0.7189	0.1097	0.2290	0.085*	
C3	0.7360 (4)	0.1241 (3)	0.4635 (7)	0.0733 (17)	
H3	0.7822	0.0902	0.5109	0.088*	
C4	0.7050 (3)	0.1624 (3)	0.5594 (6)	0.0631 (14)	
H4	0.7302	0.1550	0.6731	0.076*	
C5	0.6359 (3)	0.2127 (2)	0.4885 (5)	0.0475 (12)	
C6	0.5976 (3)	0.2576 (2)	0.5825 (5)	0.0453 (11)	
C7	0.6273 (4)	0.2469 (3)	0.7638 (5)	0.0713 (17)	
H7A	0.5935	0.2786	0.8020	0.107*	
H7B	0.6887	0.2595	0.8202	0.107*	
H7C	0.6189	0.1956	0.7856	0.107*	
C8	0.4349 (3)	0.3894 (2)	0.4339 (5)	0.0369 (10)	
C9	0.3792 (3)	0.4445 (3)	0.4806 (5)	0.0482 (12)	

### Atomic displacement parameters (Å^2^)

**Table:**

	*U*^11^	*U*^22^	*U*^33^	*U*^12^	*U*^13^	*U*^23^
Ni1	0.0409 (5)	0.0462 (5)	0.0311 (5)	0.000	0.0158 (4)	0.000
O1	0.0491 (19)	0.0503 (18)	0.0330 (16)	0.0100 (15)	0.0144 (14)	0.0013 (14)
O2	0.091 (3)	0.080 (3)	0.052 (2)	0.042 (2)	0.036 (2)	0.0173 (19)
N1	0.044 (2)	0.047 (2)	0.040 (2)	0.0055 (18)	0.0176 (18)	−0.0014 (18)
N2	0.037 (2)	0.0394 (19)	0.0332 (19)	0.0013 (18)	0.0144 (16)	0.0023 (17)
N3	0.049 (2)	0.045 (2)	0.0321 (19)	0.0077 (18)	0.0208 (18)	0.0014 (17)
N4	0.064 (3)	0.069 (3)	0.042 (2)	0.023 (2)	0.026 (2)	0.002 (2)
C1	0.058 (3)	0.065 (3)	0.052 (3)	0.008 (3)	0.028 (3)	−0.009 (3)
C2	0.069 (4)	0.075 (4)	0.065 (4)	0.019 (3)	0.025 (3)	−0.019 (3)
C3	0.062 (4)	0.072 (4)	0.072 (4)	0.032 (3)	0.016 (3)	−0.009 (3)
C4	0.058 (3)	0.067 (3)	0.051 (3)	0.020 (3)	0.010 (3)	−0.001 (3)
C5	0.045 (3)	0.051 (3)	0.043 (3)	0.004 (2)	0.015 (2)	−0.001 (2)
C6	0.043 (3)	0.053 (3)	0.036 (2)	0.004 (2)	0.013 (2)	0.004 (2)
C7	0.082 (4)	0.091 (4)	0.036 (3)	0.034 (3)	0.020 (3)	0.012 (3)
C8	0.041 (3)	0.039 (2)	0.031 (2)	−0.001 (2)	0.016 (2)	−0.001 (2)
C9	0.050 (3)	0.057 (3)	0.038 (3)	0.008 (2)	0.018 (2)	0.002 (2)

### Geometric parameters (Å, º)

**Table:**

Ni1—N2^i^	1.980 (3)	N4—H4B	0.8600
Ni1—N2	1.980 (3)	C1—C2	1.376 (6)
Ni1—O1^i^	2.074 (3)	C1—H1	0.9300
Ni1—O1	2.074 (3)	C2—C3	1.364 (7)
Ni1—N1^i^	2.094 (3)	C2—H2	0.9300
Ni1—N1	2.094 (3)	C3—C4	1.359 (7)
O1—C8	1.257 (5)	C3—H3	0.9300
O2—C9	1.225 (5)	C4—C5	1.388 (6)
N1—C1	1.333 (6)	C4—H4	0.9300
N1—C5	1.354 (5)	C5—C6	1.487 (6)
N2—C6	1.283 (5)	C6—C7	1.492 (6)
N2—N3	1.387 (5)	C7—H7A	0.9600
N3—C8	1.330 (5)	C7—H7B	0.9600
N4—C9	1.329 (5)	C7—H7C	0.9600
N4—H4A	0.8600	C8—C9	1.528 (6)
			
N2^i^—Ni1—N2	177.92 (19)	C2—C1—H1	118.3
N2^i^—Ni1—O1^i^	77.63 (12)	C3—C2—C1	118.3 (5)
N2—Ni1—O1^i^	103.84 (12)	C3—C2—H2	120.8
N2^i^—Ni1—O1	103.84 (12)	C1—C2—H2	120.8
N2—Ni1—O1	77.63 (12)	C4—C3—C2	119.5 (5)
O1^i^—Ni1—O1	93.34 (17)	C4—C3—H3	120.3
N2^i^—Ni1—N1^i^	78.26 (13)	C2—C3—H3	120.3
N2—Ni1—N1^i^	100.27 (13)	C3—C4—C5	120.3 (5)
O1^i^—Ni1—N1^i^	155.88 (12)	C3—C4—H4	119.9
O1—Ni1—N1^i^	92.02 (13)	C5—C4—H4	119.9
N2^i^—Ni1—N1	100.27 (13)	N1—C5—C4	120.4 (4)
N2—Ni1—N1	78.26 (13)	N1—C5—C6	115.2 (4)
O1^i^—Ni1—N1	92.02 (13)	C4—C5—C6	124.4 (4)
O1—Ni1—N1	155.88 (12)	N2—C6—C5	113.3 (4)
N1^i^—Ni1—N1	92.6 (2)	N2—C6—C7	125.5 (4)
C8—O1—Ni1	109.4 (2)	C5—C6—C7	121.2 (4)
C1—N1—C5	118.2 (4)	C6—C7—H7A	109.5
C1—N1—Ni1	129.2 (3)	C6—C7—H7B	109.5
C5—N1—Ni1	112.6 (3)	H7A—C7—H7B	109.5
C6—N2—N3	121.8 (3)	C6—C7—H7C	109.5
C6—N2—Ni1	120.5 (3)	H7A—C7—H7C	109.5
N3—N2—Ni1	117.6 (2)	H7B—C7—H7C	109.5
C8—N3—N2	107.9 (3)	O1—C8—N3	127.4 (4)
C9—N4—H4A	120.0	O1—C8—C9	116.4 (4)
C9—N4—H4B	120.0	N3—C8—C9	116.2 (3)
H4A—N4—H4B	120.0	O2—C9—N4	124.6 (4)
N1—C1—C2	123.4 (5)	O2—C9—C8	119.0 (4)
N1—C1—H1	118.3	N4—C9—C8	116.4 (4)
			
N2^i^—Ni1—O1—C8	177.3 (3)	Ni1—N1—C1—C2	178.8 (4)
N2—Ni1—O1—C8	−1.2 (3)	N1—C1—C2—C3	−0.4 (8)
O1^i^—Ni1—O1—C8	−104.6 (3)	C1—C2—C3—C4	0.5 (9)
N1^i^—Ni1—O1—C8	98.9 (3)	C2—C3—C4—C5	0.4 (9)
N1—Ni1—O1—C8	−2.1 (5)	C1—N1—C5—C4	1.5 (7)
N2^i^—Ni1—N1—C1	3.4 (4)	Ni1—N1—C5—C4	−178.0 (4)
N2—Ni1—N1—C1	−178.1 (4)	C1—N1—C5—C6	179.9 (4)
O1^i^—Ni1—N1—C1	−74.4 (4)	Ni1—N1—C5—C6	0.4 (5)
O1—Ni1—N1—C1	−177.2 (3)	C3—C4—C5—N1	−1.4 (8)
N1^i^—Ni1—N1—C1	81.9 (4)	C3—C4—C5—C6	−179.6 (5)
N2^i^—Ni1—N1—C5	−177.3 (3)	N3—N2—C6—C5	179.9 (3)
N2—Ni1—N1—C5	1.2 (3)	Ni1—N2—C6—C5	4.1 (5)
O1^i^—Ni1—N1—C5	104.9 (3)	N3—N2—C6—C7	0.5 (7)
O1—Ni1—N1—C5	2.1 (5)	Ni1—N2—C6—C7	−175.3 (4)
N1^i^—Ni1—N1—C5	−98.7 (3)	N1—C5—C6—N2	−2.8 (6)
O1^i^—Ni1—N2—C6	−92.3 (3)	C4—C5—C6—N2	175.5 (4)
O1—Ni1—N2—C6	177.3 (3)	N1—C5—C6—C7	176.7 (4)
N1^i^—Ni1—N2—C6	87.5 (3)	C4—C5—C6—C7	−5.0 (7)
N1—Ni1—N2—C6	−3.1 (3)	Ni1—O1—C8—N3	1.1 (5)
O1^i^—Ni1—N2—N3	91.7 (3)	Ni1—O1—C8—C9	−178.8 (3)
O1—Ni1—N2—N3	1.3 (3)	N2—N3—C8—O1	−0.1 (6)
N1^i^—Ni1—N2—N3	−88.5 (3)	N2—N3—C8—C9	179.8 (3)
N1—Ni1—N2—N3	−179.1 (3)	O1—C8—C9—O2	−8.8 (6)
C6—N2—N3—C8	−177.0 (4)	N3—C8—C9—O2	171.4 (4)
Ni1—N2—N3—C8	−1.1 (4)	O1—C8—C9—N4	171.0 (4)
C5—N1—C1—C2	−0.6 (7)	N3—C8—C9—N4	−8.9 (6)

Symmetry code: (i) −*x*+1, *y*, −*z*+1/2.

### Hydrogen-bond geometry (Å, º)

**Table:**

*D*—H···*A*	*D*—H	H···*A*	*D*···*A*	*D*—H···*A*
N4—H4*A*···O2^ii^	0.86	2.22	2.976 (5)	147
N4—H4*B*···N3^iii^	0.86	2.25	3.074 (5)	160

Symmetry codes: (ii) *x*, −*y*+1, *z*+1/2; (iii) −*x*+1, *y*, −*z*+3/2.
